# Application of a combined model with seasonal autoregressive integrated moving average and support vector regression in forecasting hand-foot-mouth disease incidence in Wuhan, China

**DOI:** 10.1097/MD.0000000000014195

**Published:** 2019-02-08

**Authors:** Jiao-Jiao Zou, Gao-Feng Jiang, Xiao-Xu Xie, Juan Huang, Xiao-Bing Yang

**Affiliations:** a Wuhan Centers for Disease Prevention and Control; b Center for Translational Medicine, Tianyou Hospital, Wuhan University of Science and Technology, Wuhan, Hubei; c National Research Institute for Health and Family Planning; d Graduate School of Peking Union Medical College, Beijing, China.

**Keywords:** hand-foot-mouth disease, incidence, prediction, seasonal autoregressive integrated moving average model, support vector regression model, time series analysis

## Abstract

Hand-foot-mouth disease (HFMD) is a serious public health problem with increasing cases and substantial financial burden in China, especially in Wuhan city. Hence, there is an urgent need to construct a model to predict the incidence of HFMD that could make the prevention and control of this disease more effective.

The incidence data of HFMD of Wuhan city from January 2009 to December 2016 were used to fit a combined model with seasonal autoregressive integrated moving average (SARIMA) model and support vector regression (SVR) model. Then, the SARIMA-SVR hybrid model was constructed. Subsequently, the fitted SARIMA-SVR hybrid model was applied to obtain the fitted HFMD incidence from 2009 to 2016. Finally, the fitted SARIMA-SVR hybrid model was used to forecast the incidence of HFMD of the year 2017. To assess the validity of the model, the mean square error (MSE) and mean absolute percentage error (MAPE) between the actual values and predicted values of HFMD incidence (2017) were calculated.

From 2009 to 2017, a total of 107636 HFMD cases were reported in Wuhan City, Hubei Province, and the male-to-female ratio is 1.60:1. The age group of 0 to 5 years old accounts for 95.06% of all reported cases and scattered children made up the large proportion (accounted for 56.65%). There were 2 epidemic peaks, from April to July and September to December, respectively, with an emphasis on the former. High-prevalence areas mainly emerge in Dongxihu District, Jiangxia District, and Hongshan District. SARIMA (1,0,1)(0,0,2)[12] is the optimal model given with a minimum Akaike information criterion (AIC) (700.71), then SVR model was constructed by using the optimum parameter (C = 100000, =0.00001, =0.01). The forecasted incidences of single SARIMA model and SARIMA-SVR hybrid model from January to December 2017 match the actual data well. The single SARIMA model shows poor performance with large MSE and MAPE values in comparison to SARIMA-SVR hybrid model.

The SARIMA-SVR hybrid model in this study showed that accurate forecasting of the HFMD incidence is possible. It is a potential decision supportive tool for controlling HFMD in Wuhan, China.

## 1 Introduction

Hand-foot-mouth disease (HFMD) is an acute intestinal virus infection disease with rashes or herpes mainly on the hands, feet, and mouth.^[1]^ It is a common intestinal viral infection which affects children of ages 5 years and younger and is most commonly caused by Enterovirus 71 (EV71) and Coxsackie virus A16 (CA16).^[2–4]^ Humans are the only natural host for these viruses that are transmitted through 2 possible routes, personal oral–fecal route and oral–oral route.^[5,6]^ The majority of HFMD afflicting children are typically benign and self-limiting, but HFMD caused by EV71 may be more likely to result in severe complications such as myocarditis, pulmonary edema, aseptic meningoencephalitis, or even death.^[7]^

HFMD is a serious health problem in the world. Large breaks associated with EV71 first reported and described in 1974,^[8]^ have been heralded in Japan since the early 1970s.^[9]^ Since then, HFMD epidemic activity has been spread throughout the Asia-Pacific region with large outbreaks. It is also a major public health problem in mainland China. HFMD affects nearly 2 million children annually in recent years and has contributed to a rise in the financial burden. The incidence of HFMD in Wuhan city exceeded 20 per 10000 persons in 2016, which was higher than the average national level. Although reported to be down in 2017, the incidence still remained high level.

Currently, several mathematical models have been widely used in infectious disease forecasting. Reliable forecasting can make people better understand the epidemic characteristics of infectious disease and prepare for intervention measures in advance.^[10]^ Among them, the autoregressive integrated moving average (ARIMA) model was the most popular model, which have been used to explore the trend of many diseases, such as tuberculosis,^[11]^ syphilis,^[12]^ influenza,^[10]^ and others. It is extension of an autoregressive (AR) model, moving average (MA) model. If the data contain no seasonality information, the ARIMA model is perfect for forecasting. Otherwise, the seasonal autoregressive integrated moving average (SARIMA) model is more suitable for forecasting than ARIMA. In addition, SARIMA model has also been used to predict the incidence of infectious diseases such as mumps,^[13]^ tuberculosis,^[14,15]^ and dengue fever.^[16]^

In general, the incidence data of infectious disease usually contain both linear and non-linear information. The ARIMA and SAIMA model can only analyze the linear information and are not suitable for analysis of the non-linear part.^[17]^ Fortunately, support vector regression (SVR) model was suitable for the non-linear fitting with great capability.^[18]^ Support vector machine (SVM) shows great capability in classification and identification in little sample and non-linear research. SVMs estimate the regression using a set of linear functions that are defined in a high dimensional space. SVM includes SVR machine and support vector classification (SVC) machine. SVR could lead to greater potential and better performance in non-linear forecasting.^[19]^ Therefore, we performed research to develop a hybrid SARIMA-SVR model to analyze the HFMD incidence data from 2009 to 2017 and predict the monthly incidence of HFMD in Wuhan, China. The fitting and forecasting performance parameters of the combined model were compared with the single SARIMA model so as to explore the optimal model. Our model will be employed to describe the future trend of HFMD more accurate and to provide reference information for HFMD control and intervention. Meanwhile, it can be used to evaluate the effect of related intervention measures.

## 2 Materials and methods

### 2.1 Ethics statement

The ethics committee of Wuhan Centers for Disease Prevention and Control approved this study.

### 2.2 Study area

**Figure 1:**
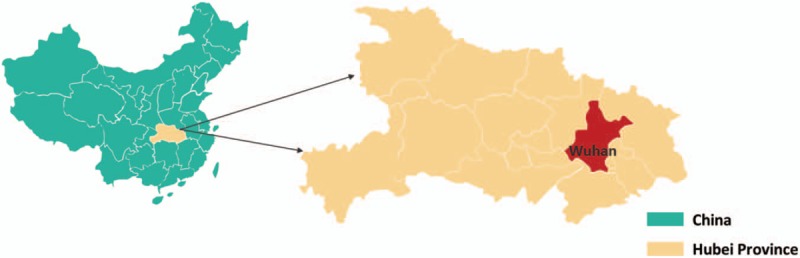
The geolocation of Wuhan City in Hubei province, China. (A) Show the geolocation of Wuhan City in Hubei province, China.

Wuhan is a central city in the Hubei Province of China, located between latitude 29°58′N and 31°22′N, and longitude 113°41 ′E and 115°05′E, in an area with a subtropical wet monsoonal climate. The city consists of 13 administrative districts with about 10 million residents, according to a demographic census in 2015, and a total land area of 8494 square kilometers. The geolocation of Wuhan City shows in Figure 1.

### 2.3 Materials

We gathered available monthly incidence data of HFMD from January 2009 to December 2017 which were reported by the China information system for disease control and prevention (CISDCP). HFMD has been statutorily notifiable since a large outbreak in May 2008. The patients were identified with HFMD according to the diagnostic criteria defined by National Health Commission of the People's Republic of China. All HFMD disease cases must be reported within 24 hours to the information system.

### 2.4 Descriptive analysis

Descriptive epidemiology method was adopted to depict the epidemical distribution of HFMD, including the temporal and spatial distribution, as well as distribution of sex, age, and occupation.

### 2.5 Data set

Data is divided into training set and test set. The training set is used to develop the models while the test set is used to evaluate the predictive validity of the models. In this study, the incidence data from 2009 to 2016 will be used as training set and the incidence data of 2017 is as test set.

### 2.6 SARIMA model

The SARIMA model is usually termed as SARIMA *(p,d,q)(P,D,Q)*_*s*_. In this expression, *p,d,q* refers to the non-seasonal order of AR, differencing, and MA respectively, while P,D,Q represent the seasonal order of differencing and MA respectively. The subscripted letter “s” indicates the seasonal period length. For example, the incidence of HFMD varies in the annual cycle, so s = 12 in this study.

The SARIMA modeling procedure consists of 3 iterative steps: identification, parameter estimation, and diagnostic checking. For large number of parameters need to calculated, the procedure of the 3 iterative steps to identify and fit a SARIMA model can be quite complex and time-consuming. Meanwhile, the procedure was performed by using the automatic algorithm with the auto.arima function in R. Therefore, in this study, we adopted the auto.arima function to develop all the 3 steps and the optimum SARIMA model was determined automatically in R3.4.3 software.

### 2.7 SARIMA-SVR hybrid model

Since the SARIMA model demonstrates professional skills in extracting the linear information from the time series data, but the residuals consist of non-linear information which the model cannot analyze. Fortunately, this information can be analyzed by the SVR model.

SVR training process is used to solving the linearly constrained quadratic programming problems that provide a unique optimal value and with no local minimum problem. Kernel function is the key factor for nonlinear mapping in SVR model. The commonly used functions are linear kernel function, radical basis function (RBF), polynomial function, multi-layer perceptron function. The type of kernel function should be set properly for it influences the parameters of SVR model. This study adopts the RBF kernel function because it suitable for most forecasting problems and it is also effective with fast training process. For the RBF kernel function, there are 3 parameters should be correctly determined in order to develop accurate estimation model:

(1) Regularization parameter *C*: *C* is parameter for determining the tradeoff cost between minimizing training error and minimizing model complexity.
(2) Kernel parameter (*γ*): *γ* represents the parameter of the RBF kernel function.
(3) The tube size of e-insensitive loss function (*ε*): *ε* is the approximation accuracy placed on the training data points.

In this study, these parameters are determined through tune.svm function in R.

So, the SARIMA-SVR hybrid model can mine the information of the data adequately by combining the advantages of the 2 basic models. We used SARIMA model to forecasting the incidence of HFMD, suppose the predicted result was 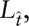 then calculated the residuals series *e*_*t*_, reconstructed the residuals series as the manipulated value to develop the SVM model, suppose the predicted result was 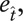 then the final prediction results are 
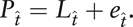
 The SVM model constructed by SVM function in R3.4.3 software.

### 2.8 Model evaluation

The predictive validity of the model was evaluated by the criteria of the mean square error (MSE) and mean absolute percentage error (MAPE) between the observed values and fitted values. 
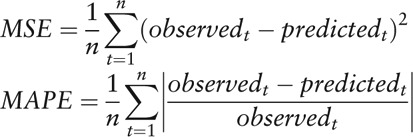


### 2.9 Statistical software

The models were performed using R3.4.3 software in this study. The MSE and MAPE were computed with Microsoft Excel software. Values of *P* <.05 were regarded as significant.

## 3 Results

### 3.1 Descriptive analysis

#### 3.1.1 Population distribution analysis

From 2009 to 2017, a total of 107636 HFMD cases were reported in Wuhan City, Hubei province, including 66195 males and 41441 females, and the male-to-female ratio is 1.60:1. HFMD mostly occurred within 0 to 5 years children, and this age group accounted for 95.06% (n = 102319) of all reported cases. The highest percentage of HFMD cases is found in scattered children, who accounted for 56.65% (n = 60981), followed by kindergarten children (accounted for 39.43%, n = 42447) and students (accounted for 3.60%, n = 3872).

#### 3.1.2 Temporal and spatial analysis

**Figure 2:**
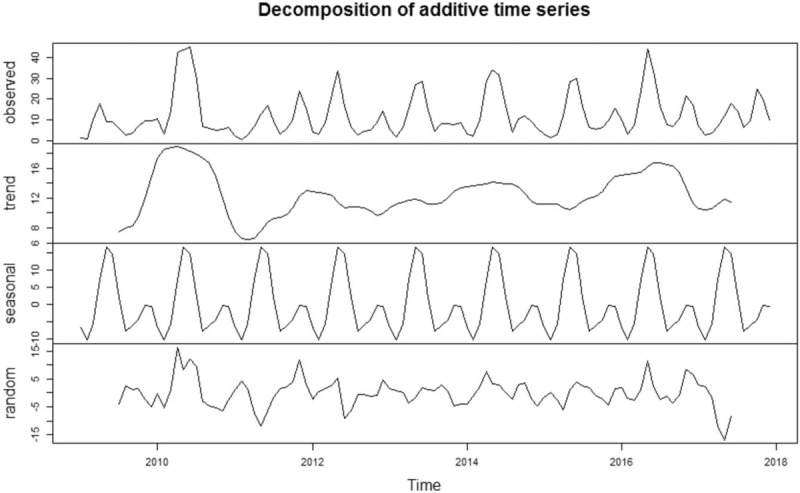
The incidence and decomposition of HFMD in Wuhan (2009–2017). (A) S how the incidence and decomposition of HFMD in Wuhan (2009–2017). (B) The seasonal part shows that though HFMD reported in every month, they also have obvious periodicity and seasonality, which shows basically as 2 epidemic peaks from April to July and September to December, with an emphasis on the former. HFMD = hand-foot-mouth disease.

The monthly HFMD incidence and decomposition are presented in Figure 2. The seasonal part shows that though HFMD reported in every month, they also have obvious periodicity and seasonality, which shows basically as 2 epidemic peaks from April to July and September to December, with an emphasis on the former.

**Figure 3:**
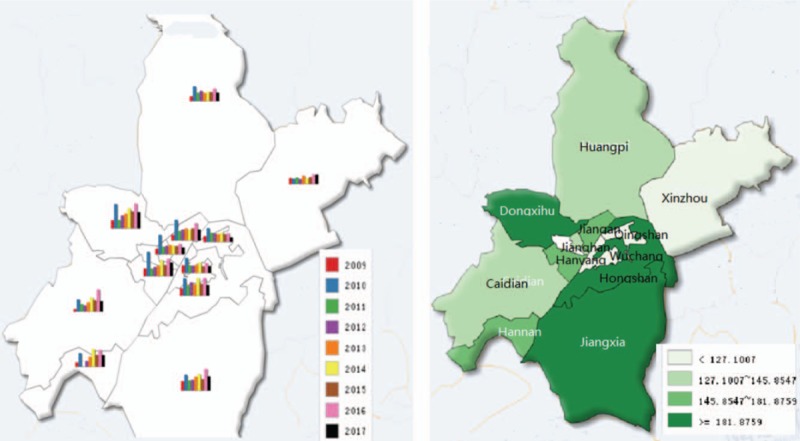
Geographical distribution and yearly HFMD incidence in Wuhan (2009–2017). (A) Show the geographical distribution and yearly HFMD incidence in Wuhan (2009–2017). (B) High-prevalence areas mainly emerge in Dongxihu District, Jiangxia District and Hongshan District. (C) Annual HFMD incidences are mapped by the histograms, the peak years for most areas in Wuhan were 2010 and 2016. HFMD = hand-foot-mouth disease.

Figure 3 shows the geographical distribution of HFMD from 2009 to 2017. High-prevalence areas mainly emerge in Dongxihu District, Jiangxia District, and Hongshan District. Annual HFMD incidences are mapped by the histograms, the peak years for most areas in Wuhan were 2010 and 2016.

### 3.2 SARIMA model

**Figure 4:**
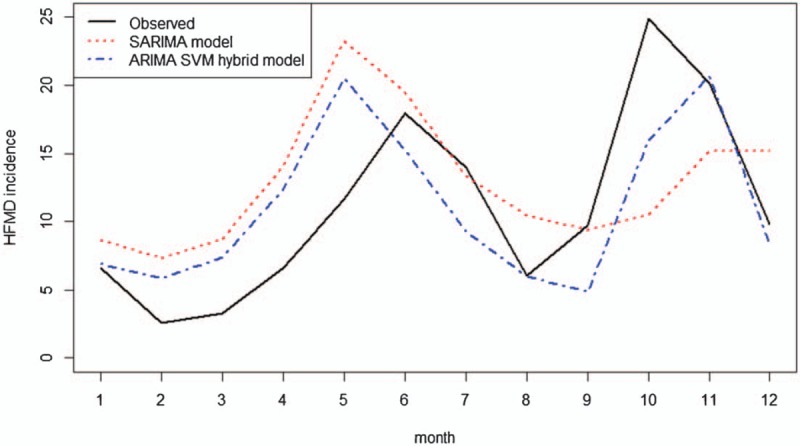
The forecasting curves of the single SARIMA model, SARIMA-SVR hybrid model and the actual HFMD incidence series in Wuhan (2017). (A) Show the forecasting curves of the single SARIMA model, SARIMA-SVR hybrid model and the actual HFMD incidence series in Wuhan (2017). (B) The forecasted incidences of single SARIMA model and SARIMA-SVR hybrid model are consistent with the changing trend of the actual incidence and matched the actual incidence well with 2 epidemic peaks from April to July and October to December. (C) The forecasted incidence of the SARIMA-SVR hybrid model fitted better than the single SARIMA model, which verified the feasibility and effectiveness of the SARIMA-SVR hybrid model. HFMD = hand-foot-mouth disease, SARIMA = seasonal autoregressive integrated moving average, SVR = support vector regression.

Training set is used to develop the model. SARIMA (1,0,1)(0,0,2)[12] is the optimal model given by auto.arima function automatically, with a minimum Akaike information criterion (AIC) (700.71). The result of the Ljung-Box *Q* test (*Q* *=* *0.08, P* *=* .78) for the model suggest the residual series is a white noise. Therefore, the SARIMA (1,0,1)(0,0,2)[12] model could extract information sufficient from the time series and is reasonable for forecasting HFMD. The forecasts of SARIMA model for 2017 HFMD are showed in Figure 4.

### 3.3 SARIMA-SVR hybrid model

The residuals of SARIMA model (Table 1) were adopted to construct SVR model. The kernel function of SVR model is RBF kernel function, the optimum parameter for SVR model obtained by tune.svm function is (C = 100000, *γ* = 0.00001, ε = 0.01). SVR model was constructed by using the optimum parameter to forecast the residuals of HFMD incidence.

**Table 1:**
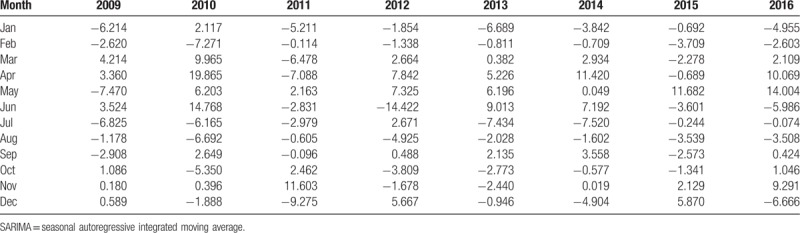
The residuals of SARIMA model.

Finally, the forecast results from SARIMA model and SVR model were combined to represent forecast results of the proposed hybrid model. Table 2 and Figure 4 shows the comparison of the actual HFMD incidence and forecasted incidence of single SARIMA model and SARIMA-SVR hybrid model in Wuhan from January to December in 2017. The forecasted incidences of single SARIMA model and SARIMA-SVR hybrid model are consistent with the changing trend of the actual incidence and matched the actual incidence well with 2 epidemic peaks from April to July and October to December. Furthermore, the forecasted incidence of the SARIMA-SVR hybrid model fitted better than the single SARIMA model, which verified the feasibility and effectiveness of the SARIMA-SVR hybrid model.

**Table 2:**
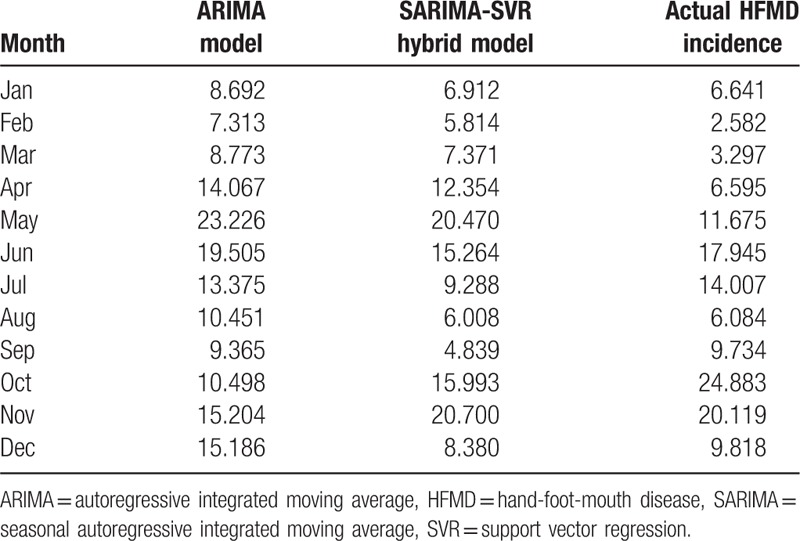
Actual value and forecasted HFMD incidence of single SARIMA model, SARIMA-SVR hybrid model (per100 000 population).

### 3.4 Model evaluation

The forecasting performance parameters of single SARIMA model, SARIMA-SVR hybrid model for the fitting and validation parts are shown in Table 3. The single SARIMA model shows poor performance with large MSE and MAPE values in comparison to SARIMA-SVR hybrid model.

**Table 3:**
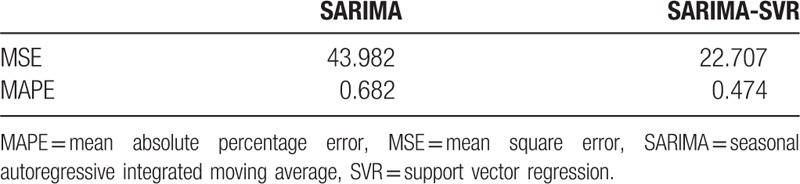
The fitting and forecasting performance of single SARIMA model, SARIMA-SVR hybrid model.

## 4 Discussion

According to our study, HFMD is one of the most serious infectious diseases in Wuhan City and affects more males than females (1.60:1) in a total of 10736 cases from 2009 to 2017, which is consistent with some previous research in other cities of China.^[2]^ The gender differences in HFMD is probably because boys are more active with a wider range of activities and more susceptible to HFMD pathogens.^[20]^ The high peaks regarding age and career of the HFMD cases were from 0 to 5 years old (95.06%) and scattered children, respectively, suggesting that the important protection ought to be focused on children.^[21]^

Furthermore, while the HFMD occurred year-round, there are 2 epidemic peaks from April to July and September to December, with an emphasis on the former, showing obvious periodicity and seasonality. Our study shows that the obvious peak incidence of HFMD appears in 2010 and 2016, which may be related to the epidemic regularity of HFMD itself, the occurrence of aggregated outbreaks and other related factors.^[22]^ Dongxihu District, Jiangxia District, and Hongshan District appear to be the high-attack areas in Wuhan City, which might be related to different demographic characteristics and vaccination coverage. The geographic differences contribute to guiding the health interventions and allocating health resources reasonably.

It is big data era now, massive data are emerging every day and permeating into almost every facet of our lives, and how to utilize the data in public health is of great significance in disease prevention and control.^[23]^ As is well known, the forecasting of infectious disease is of great significance for the prevention and control. Time series analysis of infectious disease data is useful to propose new hypotheses, anticipate epidemic trends, and improve the prevention system.^[24]^ The common time series analysis methods for disease forecast in literature reports are exponential smoothing model, artificial neural networks, ARIMA model.^[13]^

Though these methods can be used to predict the incidence trend of infectious diseases, there are some limitations respectively. For exponential smoothing model, there may be some difficulties in determining the smoothing coefficient and it could only predict at very short intervals because the weight is progressively smaller with the lengthening of the forecasting term. On the other hand, the artificial neural networks may not explain well the nonlinear functions within the time series data in practice. The ARIMA model can also be used to forecast the incidence of infectious disease, but it may not fully mining the seasonality and the nonlinear functions of the time series data.

Our study adopted a SARIMA-SVR hybrid model to predict the incidence of HFMD in Wuhan city. As one of the extended forms of the ARIMA model, the SARIMA model is particularly suitable for obvious seasonal and periodic surveillance data. We found SARIMA (1,0,1)(0,0,2)[12] is the optimal model given with a minimum AIC (700.71) in this study. Then, we calculated the residuals between the actual data and the fitted data of SARIMA model. The residuals were reconstructed to be used as the sample data to construct the SVR model. The optimum parameter is (C = 100000, *γ* = 0.00001, *ε* = 0.01), the SVR model was used to predict the residuals of HFMD incidence. Finally, we combined the results of SARIMA model and SVR model to represent the forecast results of the SARIMA-SVR hybrid model.

We compared the performance parameters of the single SARIMA model and the hybrid model by calculated the MSE and MAPE between the observed values and fitted values. The single SARIMA model shows poor performance with large MSE and MAPE values for data forecasting in comparison to SARIMA-SVR hybrid model. Therefore, the hybrid model is considered to be reasonable for forecasting with a high forecasted accuracy based on the MSE and MAPE.

Both models forecast the incidence trend accurately while the hybrid model performance better, but there is also little-observed difference between observed and forecasted values especially in May and October 2017. The reason probably due to the marketing of Enterovirus71 vaccine in October 2016 in Wuhan, the coverage of vaccine changed the population susceptibility to infection. To our knowledge, although there are a few similar studies on the HFMD in China,^[25]^ this is the first study to apply a SARIMA-SVR hybrid model for HFMD in Wuhan City. Our findings demonstrate that the hybrid model is an efficient way to forecast the dynamic change of monthly HFMD in Wuhan City, and it could be valuable to prevent and control HFMD. If the forecasted incidence is to increase, more policies or improved measures such as personal protection, and protective vaccines could be taken ahead of time. It also could be used to access the effect of the currently intervention strategies and the protective effect of the vaccine. After vaccination, the model may show that the vaccine is effective if the actual incidence is lower than the predicted result.

Although the SARIMA-SVR hybrid model showed satisfactory forecasting performance in incidence trend of HFMD, there are also some limitations should be noted. Initially, the incidence of HFMD is influenced and controlled by many factors such as the natural environment and socioeconomic factors,^[26]^ these factors were not taken into account in this study. Another limitation is that the hybrid model was merely suitable for short-term prediction.^[27]^ Hence, the model should be constantly updated in order to maintain prediction performance. Furthermore, we will explore more appropriate models to predict the incidence of HFMD in future research, such as the generalized additive model and ARIMAX model.^[9]^

## 5 Conclusion

In this study, based on the incidence data of HFMD from 2009 to 2017 in Wuhan City, we constructed the SARIMA-SVR hybrid model, which showed possibility for accurate forecasting of HFMD incidence. Our model is a potential decision supportive tool for controlling HFMD in Wuhan, China.

## Author contributions

XBY conceived and designed the experiments. JJZ analyzed the data. XXX and JH contributed reagents/materials/analysis tools. JJZ and GFJ wrote the article.

**Conceptualization:** Xiaobing Yang.

**Data curation:** Jiao-jiao Zou, Xiao-xu Xie.

**Software:** Juan Huang.

**Writing – original draft:** Jiao-jiao Zou.

**Writing – review & editing:** Gaofeng Jiang.
